# Expression Study of NDUFS1, NDUFV1, and NDUFV2 in Schizophrenia and Paranoid Personality Disorder: Role of Mitochondrial Complex I in SCZ and PPD

**DOI:** 10.31661/gmj.v11i.2165

**Published:** 2022-12-04

**Authors:** Arvin Haghighatfard, Mitra Salehi, Seyed Mehdi Saberi, Mehrdad Hashemi

**Affiliations:** ^1^ Department of Biology, North Tehran Branch, Islamic Azad University, Tehran, Iran; ^2^ Legal Medicine Research Center, Legal Medicine Organization, Tehran, Iran; ^3^ Department of Genetics, Faculty of Advanced Science and Technology, Tehran Medical Sciences, Islamic Azad University, Tehran, Iran; ^4^ Farhikhtegan Medical Convergence Sciences Research Center, Farhikhtegan Hospital Tehran Medical Sciences, Islamic Azad University, Tehran, Iran

**Keywords:** Schizophrenia, Paranoid Personality Disorder, Personality Traits, Mitochondrial Complex I, NDUFS1, NDUFV1, NDUFV2

## Abstract

**Background:**

Schizophrenia (SCZ) is a major psychiatric disorder with unclear etiology and biological diagnosis. Paranoid personality disorder (PPD) is a type-A personality disorder characterized by paranoia and generalized mistrust. The etiology and molecular mechanisms of SCZ and PPD are not clarified. The present study aimed to examine the expression alteration of three major genes of mitochondrial complex I in the peripheral blood of patients with SCZ and PPD, and its correlations with clinical features of patients, especially the five major personality traits.

**Materials and Methods:**

This case-control study was performed on 735 SCZ, 742 PPD, and 750 non-psychiatric individuals. The mRNAs level of NDUFS1, NDUFV1, and NDUFV2 were assessed using quantitative real-time polymerase chain reaction, and their correlations with psychiatric symptoms were assessed by the positive and negative syndrome scale and the brief psychiatric rating scale tests, as well as personality traits that were evaluated by NEO Five-Factor Inventory.

**Results:**

Findings showed significant overexpression of NDUFS1, NDUFV1, and NDUFV2 in patients with SCZ (P=0.001, P=0.002, and P=0.004, respectively) and PPD (P=0.001, P=0.003, and P=0.006, respectively) compared with non-psychiatrists. In addition, these genes were associated with positive psychiatric symptoms and neuroticism in SCZ (P=0.008) and PPD (P=0.01).

**Conclusion:**

Overexpression genes that encode subunits of complex I play an important role in SCZ and PPD etiology and severity of symptoms. It may bring evidence about the significant role of bioenergetics dysfunction in psychotic behaviors in different psychiatric situations.

## Introduction

Schizophrenia (SCZ) is a major chron ic neuropsychiatric disorder with al most 1% prevalence worldwide. The etiology of SCZ is unknown, and diagnosis depends only on descriptive symptomatic and psychiatric interviews. SCZ shows great symptomatic heterogeneity in the presence and severity of positive, negative, and general symptoms, which lead to many complexities in the diagnosis and treatment of patients [1][2]. Positive symptoms include hallucinations, delusions, and paranoia; negative symptoms include affective flattening, lack of motivation, poor speech, social withdrawal, cognitive impairments (i.e., attention deficits and disrupted memory functions), and severe impairments in executive functions [3]. Clinical symptoms of SCZ, mostly onset during adolescence or early adulthood may support the pieces of evidence about neurodevelopmental disturbance's role in its pathophysiology. SCZ and related complex psychiatric disorders may represent the endpoint of several different pathogenic pathways [4]. Post-mortem studies of SCZ could strengthen the neurodevelopmental model due to detected pre-existing morphological abnormalities in the brains of patients with SCZ at the onset of the condition [5]. In addition, reports show that SCZ demonstrates several behavioral abnormalities and executive function deficiencies in childhood years before the onset of symptoms [6].

Paranoid personality disorder (PPD) is a type-A personality disorder characterized by paranoia and pervasive, long-standing suspiciousness, and generalized mistrust of others [7]. The prevalence of PPD was estimated at 0.5% to 2.5% in the general population in different countries [8]. No clear etiology or molecular mechanism was suggested for PPD, but the heritability of this disorder is high [9]. The first molecular genetics study on PPD detected several shared genetic biomarkers between SCZ and PPD in mitochondrial complex I [10].

Evidence indicates that SCZ has pathological components, which can be attributable to the abnormalities of mitochondrial function [11]. Also, it has been reported that mitochondrial dysfunctions negatively affect neuronal plasticity and cause cognitive deficits, behavioral abnormalities, and executive dysfunction in several neurologic and neuropsychiatric disorders, including Parkinson, bipolar disorder, and SCZ [11].

Several studies have examined the expression of mitochondrial complex genes in brain tissues, lymphocytes, and whole blood of patients with SCZ [12]. In post-mortem studies, expression results varied in different locations of the brain tissue [1][3]. BenShachar and Karry reported an increase in the enzymatic activity of complex I in the peripheral tissues of patients with SCZ [1]. Dror et al. [6] reported that the overexpression of mitochondrial complex I genes in patients with SCZ was associated with positive and negative symptoms. The study of mitochondrial complex1 genes has been repeated in European societies by Mehler-Wex et al. among SCZ patients and detected overexpression of NDUFS1(encoding the greatest subunit of mitochondrial complex1) as a marker for SCZ [2]. It seems that clinical observations and the correlation of expression profile with psychiatric symptoms were almost missing points of these studies. Akarsu et al. studied the correlation of four genes' expression in complex I and III with psychiatric tests resulting in different subtypes of SCZ and duration of illness [9]. They confirmed the overexpression of NDUFS1, NDUFV1, and NDUFV2 in blood. They also found a positive correlation of NDUFV2 gene overexpression with the brief psychiatric rating scale (BPRS) and simplified acute physiology score in the first episode of SCZ [9]. Therefore, studying mitochondrial dysfunction mechanisms in SCZ may help better understand the etiology and new diagnostic markers.

The present study aimed to comprehensively investigate the correlation of transcripts levels of three genes that encode important mitochondrial complex I subunits with the severity of psychiatric symptoms of SCZ and PPD. Also, we evaluated the five major personality traits in all participants and the correlation between them.

## Materials and Methods

**Table:** Table2. Clinical and Demographic Characteristics of Subjects

**Genes**	**Primer sequence**
***NDUFS1***	F: 5'TGTGCCTTGTTGAAATTGAGAAAG3'
	R: 5'GCATAGGGCTTAGAGGTTAGGG3'
***NDUFV1***	F: 5'TACATCCGAGGGGAATTCTACA3'
	R: 5'GTTCTTTCAAGGGCACAGACAT3'
***NDUFV2***	F: 5'GGAGGAGCTTTATTTGTGCAC3'
	R: 5'CCTGCTTGTACACCAAATCC3'
***β-actin***	F: 5'TGAAGTGTGACGTGGACATCCG3'
	R: 5'GCTGTCACCTTCACCGTTCCAG3'

### 1. Subjects Selections and Ethical Issues

This case-control study was conducted in the molecular laboratory of the North Tehran Branch, Islamic Azad University, Tehran, Iran, from February 2019 until January 2020. We enrolled 735 SCZ, 742 PPD, and 750 nonpsychiatric individuals. Two independent senior psychiatrists diagnosed all patients based on extended clinical interviews and the Diagnostic and Statistical Manual of Mental Disorders fifth edition (DSM-5) chart review. All patients received treatment, including antipsychotic medications, during the 18- to 24- months period before sampling and were considered as the chronic disease. Patients were selected from psychiatric hospitals and outpatient clinics. Patients with co-morbid psychiatric disorders and schizoaffective or schizotypal disorders were excluded. The estimated daily dose average of antipsychotics was calculated using chlorpromazine (CPZ) equivalents; and estimated lifetime CPZ equivalents considering the duration of the illness. The non-psychiatric group was matched for demographic parameters (such as sex, age, race, socioeconomic situation, familial situation, and education level) with both patient groups and with no history of any psychological problems, no current and/or history of severe medical conditions, neurological disorder, history of head trauma with loss of consciousness, no drug abuse, and alcohol and/or nicotine dependence [2][5].

Before the beginning of the study, written informed consent was provided according to the declaration of Helsinki. Also, the study was approved by the ethical committee of the North Tehran Branch, Islamic Azad University, Tehran, Iran (code number: 42640).

### 2. Sampling and Gene Expression Evaluation

Blood samples (5 ml) were collected from the cubital vein without a tourniquet using PAXgene blood RNA tubes (PreAnalytiX,Hombrechtikon, Switzerland) between 10 and 11 A.M, and RNA extraction started immediately after sampling. Total RNA was extracted from peripheral blood samples according to the column-based standard protocols of the RNA purification kit (GeneJETTM, Fermentas, Latvia). Total RNA was treated with DNase to prevent the contaminating of genomic DNA using DNase treatment and removal reagents (DNase I, Fermentas, Latvia), according to the manufacturer's protocol. The quality and integrity of extracted RNA were examined by gel electrophoresis and ultraviolet spectroscopy with a Nanodrop 1000 spectrophotometer (Thermo Scientific, Wilmington, DE, USA), and sampling was repeated for subjects with low-quality RNA in the first sample. Then cDNA was synthesized with a transcription first strand cDNA synthesis kit (RevertAid Premium First Strand cDNA Synthesis Kit, Fermentas, Latvia) according to the manufacturer’s protocol. Primers for reference and interest genes were designed by ‘oligo7’ software, blasted on the NCBI website, and checked in In-Silico polymerase chain reaction (PCR) of the UCSC Genome Browser website (available at: https://genome.ucsc.edu/). PCR and agarose gel electrophoresis were used to verify the predicted size of PCR amplicons of genes.

Standard curves for each gene were prepared using serial dilutions (1:4) of pooled cDNA from total RNA extracted from blood samples of 10 non-psychiatric subjects. In each experiment, the R2 value of the standard curve was more than 0.99, and no-template control assays resulted in no detectable signal. Quantitative real-time PCR (qPCR) was performed using SYBR green (Thermo Scientific Maxima SYBR Green/ROX qPCR Master Mix [2X], Fermentas, Latvia). A triplicate method was performed for qPCR using a 7900HT Fast Real-Time PCR System with a Fast 96-Well Block Module (Applied Biosystems, Foster City, CA, USA). PCR data were obtained by Sequence Detector Software (SDS; version 2.3 Rev C Patch, Applied Biosystems, Foster City, CA, USA) and quantified by the standard curve method. SDS software plotted the real-time fluorescence intensity, selected the threshold within the linear phase of the amplicon profile, and drew a standard cycle curve at the threshold versus extracted RNA quantity. Samples were measured in one plate for one target gene, and their Cq values were in the linear range of the standard curve. In qPCR tests, outliers or sample failures were repeated for each gene. The ratio was calculated using the Pfafle formula. Normalization of the qPCR experiment was conducted by the beta-actin gene as an endogenous reference gene. Blood sampling and gene expression processes were performed based on previous studies [13][14][15][16]. Primers of reference and target genes are presented in Table-1.

### 3. Psychiatric Symptoms Examinations and Psychological Assessments

#### 3.1. Positive and Negative Syndrome Scale (PANSS)

Symptoms severity was measured with PANSS. The PANSS is a well-known psychiatric test with a 30-item semistructured interview that assesses three major symptom categories associated with SCZ (positive, negative, and general symptoms). Seven positive, seven negative, and sixteen general symptoms have been measured on the PANSS rating scale [17]. PANSS tests were obtained from SCZ and PPD patients by a senior psychiatrist.

### 3.2. BPRS

The BPRS test is a 24-item rating scale for

the evaluation of the severity of psychiatric symptoms such as depression, anxiety, hallucinations, and unusual behavior [18]. The correlation between each symptom scale and differentially expressed genes and pathways was calculated similarly to an analysis conducted for the PANSS test. Based on previous studies, the BPRS and PANSS tests were scored on all live patients with Meth-induced psychoses and SCZ [19].

### 4. NEO Five-Factor Inventory (NEO-FFI)

A revised version of the NEO-FFI is a well-known psychological personality inventory that consists of 240 questions intended to assess the big five personality traits, including extraversion, agreeableness, conscientiousness, neuroticism, and openness to experience. A shortened version of the NEO-FFI was used in the present study to evaluate these domains. NEO-FFI test contains 60 items, including 12 items for each domain [20]. In the present study, NEO-FFI results were presented by T score after the interpretation by a senior psychologist.

### 5. Statistical Analysis

Normal distribution for continuous variables was checked via the KolmogorovSmirnov test. Multiple group comparisons were calculated by one-way ANOVA and independent Student's t-test. The Pearson correlation test indicated the relationship between clinical and genetic variables, and the Bonferroni correction test was used for multiple comparison corrections. Also, Cohen's kappa coefficient to measure inter rater reliability. Statistical analysis was conducted by using the SPSS version23 (IBM Corporation, Armonk, New York, USA). Statistical significance was set at P<0.05.

## Results

**Table:** Table2. Clinical and Demographic Characteristics of Subjects

**Variables**	**SCZ**	**PPD**	**Non-psychiatric**	**P-value**
**Gender, n**				
Male	375	380	385	0.82
Female	360	362	365	
*Age, y	31 (25 to 39)	32 (27 to 38)	31 (25 to 32)	0.78
*Age of onset, y	21 (17 to 24)	20 (18 to 29)	-	0.2
*PANSS score	77.22 (66 to 76)	64.11(56 to 70)	-	0.04
*BPRS score	56.28 (44 to 57)	51.29 (37 to 59)	-	0.042
**NEO-FFI**				
*Neuroticism	64 (61 to 68)	62 (57 to 67)	39 (31 to 42)	0.001
*Extraversion	43 (41 to 55)	39 (37 to 47)	58 (55 to 59)	0.01
*Openness	44 (40 to 57)	42 (39 to 56)	59 (53 to 59)	0.01
*Agreeableness	43 (41 to 50)	40 (36 to 44)	55 (54 to 61)	0.02
*Conscientiousness	42 (38 to 48)	41 (35 to 47)	56 (53 to 62)	0.02

^*^Data presented as mean with lowest and highest range. **PPD:** Paranoid personality disorder; **SCZ:** Schizophrenia; **PANSS:** Positive and negative syndrome scale; **BPRS:** Brief psychiatric rating scale; **NEO-FFI:** NEO five-factor inventory

**Table:** Table3. Comparison of NEO-FFI Test Results between Groups.

**Personality traits**		**P-Value**	
	**SCZ vs. non-psychiatric**	**PPD vs. non-psychiatric**	**SCZ vs. PPD**
**Neuroticism**	0.003	0.003	0.2
**Extraversion**	0.01	0.008	0.14
**Openness**	0.01	0.01	0.33
**Agreeableness**	0.01	0.009	0.21
**Conscientiousness**	0.01	0.01	0.15

**SCZ:** Schizophrenia; **PPD:** Paranoid personality disorder;

**Table:** Table4. Gene Expression Ratio for Each Group

**Genes**	**SCZ vs. non-psychiatric**	**PPD vs. non-psychiatric**
***NDUFS1***	1.77 (95%CI: 1.44 to 1.88)	1.49 (95%CI: 1.46 to 1.92)
***NDUFV1***	1.63 (95%CI: 1.43 to 2.11)	1.81 (95%CI: 1.46 to 1.96)
***NDUFV2***	1.92 (95%CI: 1.37 to 2.37)	1.73 (95%CI: 1.48 to 2.46)

**SCZ:** Schizophrenia; **PPD:** Paranoid personality disorder; **CI:** Confidence interval

Demographic and clinical data are presented for each group in Table-2. Individuals with SCZ and PPD showed significant differences in all five personality traits compared with the non-psychiatric group. Statistical analysis of NEO-FFI results between groups is presented in Table-3.

### Gene Expressions

There were significant differences in complex

I gene mRNA levels between the two patient groups and controls. The expression level of NDUFS1 (P=0.001), NDUFV1 (P=0.002), and NDUFV2 (P=0.004) were up-regulated in the SCZ group compared with controls. Also, Expression levels of NDUFS1 (P=0.001), NDUFV1 (P=0.003), and NDUFV2 (P=0.006) up-regulated in the PPD group compared with controls. Results of the gene expression ratio are presented in Table-4.

### Correlation Between mRNA Levels of Mitochondrial Complex I Genes and Clinical Characteristics

There was no significant correlation between expression results of any genes and sex, race, age, and age of onset of the two patients group. Also, the duration of disease in the SCZ group was positively correlated with overexpression of NDUFS1 (P=0.005) and NDUFV1 (P=0.007).

### Correlation Between mRNA Levels of Mitochondrial Complex I Genes and Psychiatric Tests Results

There was a significant positive correlation between overexpression of NDUFS1, NDUFV1, and NDUFV2 and total scores of PANSS and BPRS in patients with SCZ. Also, we found a significant positive correlation between PANSS scores and NDUFS1 expression (P=0.003). For the BPRS score, a significant positive correlation between NDUFV1 (P=0.02) and NDUFS1 (P=0.006) was detected.

### Correlation Between mRNA Levels of Mitochondrial Complex I Genes and Personality Traits

There was a significant positive correlation between overexpression of NDUFS1 and neuroticism in SCZ (P<0.001) and PPD (P<0.01) groups.

## Discussion

Gene expression is a language that shows the patterns of the molecular basis and the current situation of disorders, especially multifactorial disorders. Also, gene expression results with the combination of clinical features can help with reclassification, early diagnosis, a better understanding of etiology, and following the treatment progresses in disorders. As all of these parameters are still unclear in SCZ and PPD, we decided to study the expression of three important genes of mitochondrial complex I, along with clinical and psychological assessments.

Overexpression of NDUFS1, NDUFV1, and NDUFV2 and their positive correlation with psychiatric symptoms and abnormalities in personality traits were detected in both SCZ and PPD individuals.

Overexpression of NDUFS1 (75kDa subunit), NDUFV1 (51kDa subunit), and NDUFV2 (24kDa subunit) has been reported in SCZ [5]. Several genes in essential components complex I, such as NDUFA1 (7.5kDa subunit) and NDUFB11 (17.3kDa subunit) have been reported in progressive neurodegenerative diseases, e.g., Alzheimer [11][13]. The NDUFB11 mutation was reported in mitochondrial encephalomyopathy and down expression of NDUFB11 in human postmortem brain tissue samples of patients with SCZ using RNA-seq [21][22]. NDUFS1, NDFV1, and NDUFV2 genes overexpression may support the hypothesis of the similarity of PPD and SCZ pathophysiology and molecular pathway related to psychotic behaviors. Antipsychotics' target pathways are dopaminergic and serotonergic. The previous studies showed that mitochondrial function was modulated by dopamine inhibition of respiratory complex I activity [23]. Therefore, gene expression alternation in mitochondrial complexes may affect drug pharmacodynamics responses. Further genetic studies on antipsychotic resistance could lead to the prediction of treatment responses in psychiatric disorders.

The correlation between the total score of BPRS, PANSS, and genes' expression changes indicated SCZ as a progressive neurologic disorder. However, subscales of these tests may show more specific information. The correlation of positive symptoms scores of PANSS and BPRS scores with overexpression of the NDUFS1 gene-the gene that is considered in paranoid subtype and PPDs-may determine this gene as a strong marker for paranoid thoughts, paraphrenia, and delusional disorder [24][25]. Mitochondria and several cellular components lead to constant crosstalk, modulating transcriptional, and post-translational processes. Mitochondrial dysfunctions and their potential effects on genome-wide regulation of gene expression have been considered a major cause of the etiology and severity of symptoms in SCZ and PPD [26].

The present study examined the correlations between personality traits among patients with SCZ and PPD with mitochondrial complex I functions. Our results confirmed previous reports about the positive correlation between neuroticism and psychotic experiences, while extraversion, openness, agreeableness, and conscientiousness were negatively correlated [27]. These findings suggest a relationship between personality traits and psychotic experiences. In addition, the correlation of NDUFS1 mRNA level with an increase in neuroticism may provide evidence about the significant role of the 75kDa subunit in neuron functions that affect neuroticism traits.

The sample size of PPD subjects in the present study compared with previous studies may increase the importance of the findings. On the other hand, personality and clinical assessments could clarify the role of mitochondria in neuron functions and turn the psychological situation of individuals. The lack of accessibility to brain tissue is an obstacle to understanding the exact role of gene expression alterations of mitochondrial genes in neuron functions and psychiatric

situations. The lack of neuroimaging evaluations was the most important limitation of the clinical part of the present study. Also, the lack of protein level confirmation for gene expression analysis was another limitation of the current study.

## Conclusion

Our findings present three genes in the respiratory chain involved in SCZ and PPD. Present results may explain the psychosis of PPD and SCZ as a neurodegenerative phenomenon. These correlations between psychiatric symptoms and gene expressions present them as a novel possible marker in SCZ and PPD and other neurodevelopment neurodegenerative disorders. Personality assessments may suggest these genes as potential markers for personality traits such as neuroticism as well as disorders. Longterm gene expression studies and more comprehensive clinical and neurological studies could help find more subtypes' specific markers and the pathogenesis of psychiatric disorders and reclassify them based on affected pathways.

## Conflict of Interest

The authors declare that they have no conflict of interest.
